# Detecting controlling nodes of boolean regulatory networks

**DOI:** 10.1186/1687-4153-2011-6

**Published:** 2011-10-11

**Authors:** Steffen Schober, David Kracht, Reinhard Heckel, Martin Bossert

**Affiliations:** 1 Institute of Telecommunications and Applied Information Theory, Ulm University, Ulm, Germany; 2 The Communication Technology Laboratory, ETH Zürich, Switzerland

## Abstract

Boolean models of regulatory networks are assumed to be tolerant to perturbations. That qualitatively implies that each function can only depend on a few nodes. Biologically motivated constraints further show that functions found in Boolean regulatory networks belong to certain classes of functions, for example, the unate functions. It turns out that these classes have specific properties in the Fourier domain. That motivates us to study the problem of detecting controlling nodes in classes of Boolean networks using spectral techniques. We consider networks with unbalanced functions and functions of an average sensitivity less than $\frac{2}{3}k$, where *k *is the number of controlling variables for a function. Further, we consider the class of 1-low networks which include unate networks, linear threshold networks, and networks with nested canalyzing functions. We show that the application of spectral learning algorithms leads to both better time and sample complexity for the detection of controlling nodes compared with algorithms based on exhaustive search. For a particular algorithm, we state analytical upper bounds on the number of samples needed to find the controlling nodes of the Boolean functions. Further, improved algorithms for detecting controlling nodes in large-scale unate networks are given and numerically studied.

## 1 Introduction

The reconstruction of genetic regulatory networks using (possibly noisy) expression data is a contemporary problem in systems biology. Modern measurement methods, for example, the so-called *microarrays*, allow measuring the expression levels of thousands of genes under particular conditions. A major problem is to predict the structure of the underlying regulatory network. The overall goal is to understand the processes in cells, for example, how cells execute and control operations required for the functions performed by the cell. In the Boolean model, this implies that based on a given set of observed state-transition pairs (samples), the Boolean functions attached to each node need to be identified. In general, this problem is quite hard, due to the large number of possible Boolean functions. First results for the noiseless case appeared 1998 in the work of Liang et al. [1]. Their *Reverse Engineering Algorithm *(REVEAL) tries in a first step to find the *controlling nodes *of each node by estimating the mutual information between possible variables and the regulatory function's output. After the inputs have been identified, the *truth table *of the Boolean functions can be determined from the samples. If the number of variables for each function is at maximum *K*, the REVEAL algorithm considers any of the $\left({}_{K}^{n}\right)$ combinations of variables, where *n *is the number of nodes in the network.

The numerical results in [1] suggest that it is possible to identify a Boolean network using a small number of samples. Akutsu et al. [2] gave an analytical and constructive proof that it is possible to identify the network using only O(logn) samples with high probability. For constant values of *K*, the given algorithm, BOOL, has *time complexity *$O\left(n^{K+1}\cdot m\right)$ where *m *is the number of samples. Later it was shown that a similar algorithm also works in the presence of (low-levela) noise [3]. These algorithms are based on exhaustive search in two ways. First, they search through all $\left({}_{K}^{n}\right)$ possible combinations of controlling nodes. Second, they search through all of the $2^{2^{K}}$ possible Boolean functions. Lähdesmäki et al. [4] overcame the problem to search through all possible Boolean functions, reducing the double exponential factor to roughly 2*^K^*. But their algorithm still searches through all $\left({}_{K}^{n}\right)$ possible variable combinations, hence, runs roughly in time *n^K^*. If *n *is large, applying such an algorithm is prohibitive even for moderate values of *K*.

The algorithms above implicitly solve two distinct problems. First, the controlling nodes of all nodes have to be detected, and second, each function has to be determined. This paper is dedicated to algorithms for detecting controlling nodes in Boolean networks. In general, this problem can be solved by exhaustive search in time *n^K^*. By exploiting structural properties of certain classes of functions, the time and *sample complexity *of the algorithms can be reduced. The sample complexity of an algorithm is the number of samples needed to detect the controlling nodes with a predefined probability. In fact, one can readily apply methods stemming from the area of PAC (probably approximately correct) learning theory [5], as the network identification problem can be reduced to the problem of learning Boolean *juntas*, i.e., Boolean functions that depend^b ^only on a small number of their arguments. This problem was studied by Arpe and Reischuk [6] extending earlier work of Mossel et al. [7,8].

The particular inference problem studied here is the following. Given a synchronous Boolean network and a set of input/output patterns, i.e.,

$$\left\{\left(X_{1}^{\prime },Y_{1}^{\prime }\right),\left(X_{2}^{\prime },Y_{2}^{\prime }\right),\ldots ,\left(X_{m}^{\prime },Y_{m}^{\prime }\right)\right\},$$

where $X_{l}^{\prime }$ and $Y_{l}^{\prime }$ describe noisy observations of two successive network states **X***_l _*and **Y***_l _*at some time *t_l _*and *t_l _*+ 1, respectively. The networks state **X***_l _*at time *t_l _*is modeled using a uniformly distributed random variable **X**.

The task to detect the controlling nodes can be reduced to the problem to find the *essential variables *of the Boolean functions. This problem is easier to solve for some classes of functions, namely for nearly all unbalanced functions and functions of an average sensitivity less then $\frac{2}{3}k$, where *k *is the number of controlling variables for a function. Further the class of 1-low networks, which include unate networks, linear threshold networks, and networks with nested canalyzing functions, is considered. The application of spectral learning algorithms leads to both better time and sample complexity for the detection of controlling nodes compared with exhaustive search. In particular, a slight improvement in the algorithm given in [6] is presented, for which analytical bounds on the number of samples needed to find the controlling nodes are derived. It is notable that for the class of 1-low networks, the time complexity of the resulting algorithms is roughly *n*^2^. The algorithm is further improved, where the main focus lies on the identification of controlling nodes in a large-scale unate network.

Finally, the performance of the improved algorithms is evaluated for large-scale unate networks with 500 nodes using numerical simulations. Further, the problem is studied in a Boolean network model of a control network of the central metabolism of *Escherichia coli *with 583 nodes [9]. Preliminary results of this work were presented in [10,11].

The outline of the paper is as follows. In Section 2, Boolean networks are defined and the detection problem is formally stated. The two classes of functions considered here are introduced and discussed. Section 3 gives a brief introduction to the Fourier analysis of Boolean functions and discusses the spectral properties of the two classes of functions. Further, the algorithms are stated and analyzed in 3.3 and 3.4. Simulation results are presented in 3.5.

## 2 Regulatory networks and inference

### 2.1 Boolean regulatory networks

A Boolean network (BN) of *n *nodes can be described by a numbered list *F *= {*f*_1_, *f*_2_, ..., *f_n_*} of Boolean functions (BFs) *f_i _*: {-1, +1}*^n ^*→ {-1, +1}. Each node *i *in the network has a binary state variable *x_i_*(*t*) ∈ {-1, +1} assigned, which may vary in time *t *∈ ℕ. The networks state at time *t *is given by **x**(*t*) = (*x*_1_, *x*_2_, ..., *x_n_*)(*t*) ∈ {-1, +1}*^n^*. The state of a node *i *at time *t *+ 1 is given as

$$x_{i}\left(t+\text{1}\right)=f_{i}\left(x\left(t\right)\right),$$

i.e., given by the pre-state of the network **x**(*t*) and the Boolean functions *f_i_*.

In general not all of the possible *n *variables of a function *f_i _*are *essential*. The *i*th variable is called essential to *f *if and only if there exists at least one **x **∈ {-1, +1}*^n ^*such that *f*(*x*_1_, ..., *x_i_*, ..., *x_n_*) ≠ *f*(*x*_1_, ..., -*x_i_*, ..., *x_n_*). An equivalent terminology is that the function *f depends *on the *i*th variable. For any function *f*, the set var(*f*) ⊆ {1, ..., *n*} is defined by

i∈var (f) if and only if the ith variable is essential to f;

hence, var(*f*) is called the *set of essential variables *of *f*. If var(*f*) ≤ *k*, a function *f *with *n *variables is usually called a (*n*, *k*)-junta.

Finally note that each BN can be associated with a directed graph that allows describing the network using graph theoretic terms. Let *G*(*V*, *E*) be a directed graph, where *V *= {1, 2, ..., *n*} is the set of nodes and *E *⊆ *V *× *V *is the set of edges. The set *E *is defined by

$$\left(i,j\right)\in E\;\text{if and only if}\;i\in \text{var}\left(f_{j}\right).$$

### 2.2 The detection problem

Assume that there exists an unknown BN that is an appropriate description of an underlying dynamical process, for example, a regulatory network. An experiment generates state-transition pairs by observing the process, but in general, the *measurements *of the state-transitions are noisy. The challenge is now to detect the functional dependencies between the nodes of the network.

This problem can be restated as follows: Assume that a function *f *is chosen at random from a subset of functions . A single state-transition contains a pre-state **X***_l _*∈ {-1, +1}*^n^*, chosen according to a well defined distribution and the corresponding output of the function Y*_l _*= *f*(**X***_l_*). Each component X_*l*, *i *_and Y*_l _*is independently flipped with probability *ϵ*. In the following, *ϵ *is called the noise rate. In this way, a set of *m *noisy observations or samples,

$$X^{m}=\left\{\left(X_{1}^{\prime },Y_{1}^{\prime }\right),\left(X_{2}^{\prime },Y_{2}^{\prime }\right),\ldots ,\left(X_{m}^{\prime },Y_{m}^{\prime }\right)\right\},$$

is obtained. In the following, it is assumed that **X **is uniformly distributed. Some comments on choosing **X **uniformly distributed will be given in the last section. Given a set of samples, the task is to detect the set of essential variables of *f*. This should be achieved in an efficient way, since the number of nodes can be very large in realistic problems. Further, the probability of a *detection error *should be as small as possible.

### 2.3 Classes of regulatory functions

Different classes of functions have been proposed to model regulatory functions. The authors do not attempt to interfere in this discussion. Merely, the approach taken here is to show that many of the proposed functions fall into two classes for which Fourier-based algorithms provide an advantage in running time over algorithms based on exhaustive search. A precise definition is given later. Two classes of functions that may be reasonable models of functions in genetic regulatory networks are presented. For both of these classes, it is assumed that the number of essential variables is less or equal to *k*. The first class, denoted by $C_{\left\lceil \frac{2}{3}k\right\rceil }$, includes

• functions with average sensitivity less than $\frac{2}{3}k$, and

• unbalanced functions,

where it is assumed that for any function *f *any restriction *f′ *on *k′ *> 1 of its essential variables has an average sensitivity less or equal than $\frac{2}{3}k^{\prime }$ or is an unbalanced functions (or both). Note that a restriction *f′ *is obtained from *f *by setting some of its variables to fixed values. The second class $C_{1}$ includes

• unate functions, which further include

- nested canalizing functions, and

- linear threshold functions.

The average sensitivity of a Boolean function *f *is defined as

$$\text{as}\left(f\right)=\underset{i}{\sum }{\text{I}}_{i}\left(f\right),$$

where I*_i_*(*f*) is the *influence *of the variable *i *on *f*, [12], defined as

(1) $${\text{I}}_{i}\left(f\right)\;=\text{ Pr }\left\{f\left(X_{\text{1}},\;.\;.\;.\;,X_{i},\;.\;.\;.\;,X_{n}\right)\neq f\left(X_{\text{1}},\;.\;.\;.\;,-X_{i},\;.\;.\;.\;,X_{n}\right)\right\}.$$

Basically, low average sensitivity is a prerequisite of non-chaotic behavior in random Boolean networks (RBNs), in particular, the expectation of the average sensitivity has to be less or equal to 1 [13]. This motivates to study the class $C_{\left\lceil \frac{2}{3}k\right\rceil }$ as it is widely assumed that Boolean models of biological networks are tolerant to perturbations. Unbalanced functions^c ^are of interest due to a similar reason; namely, it is well known that the average sensitivity of balanced functions is lower bounded by 1 [14]. Hence, a function that has average sensitivity less than 1 is necessarily unbalanced.

Unate functions were shown to be of interest in the biological context by Grefenstette et al. [15]. These functions arise as a consequence of a biochemical model. They can be defined in terms of monotone functions. A function *f *is called monotone if *f*(**x**) ≤ *f*(**y**) holds for every **x **≤ **y**, where **x **≤ **y **⇔ *x_i _*≤ *y_i_*. A function *f*(**x**) = *f*(*x*_1_, *x*_2_, ..., *x_n_*) is said to be unate if there exists some fixed *σ *∈ {-1, +1}*^n ^*such that *f*(*x*_1_·*σ*_1_, *x*_2_·*σ*_2_, ..., *x_n_*·*σ_n_*) is a monotone function. Besides the results of Grefenstette et al., the class of unate functions is considered to be very promising because each variable of a unate function is correlated with its output. This property was conjectured to be important from the first days on [1]. Secondly, it contains the class of nested canalyzing functions and linear threshold functions which can often be found in Boolean models of regulatory networks. Kauffman et al. [16] discussed nested canalizing functions in the context of RBNs and found them to have a stabilizing effect on the networks. Notably, Samal et al. [17] reported that in the large-scale Boolean model of the regulatory network of the *E*. *coli *metabolism [9], the input functions of 579 out of 583 genes are, at least, canalyzing. Further investigations by the authors of the present paper revealed that all functions are unate. Linear threshold functions (LTFs) often appear in Boolean models of regulatory networks, for example, [18,19]. A Boolean function is a LTF if it can be represented by

$$f\left(x_{1},x_{2},\ldots ,x_{n}\right)=\left\{\begin{array}{cc} +1 & \text{if}\;w_{0}+\sum_{i=1}^{n}w_{i}\cdot x_{i}\geq 0\\ -1 & \text{otherwise} \end{array}\right),$$

where *w_i _*∈ ℝ. For *n *< 4, the classes of unate and linear threshold functions coincide [20].

## 3 Learning essential variables of regulatory functions

### 3.1 Fourier analysis and learning

Let *f *: {-1, 1}*^n ^*→ {-1, 1} be a *n*-ary BF. Any function *f *can be represented by its *Fourier *expansion

(2) $$f\left(x\right)=\underset{U\subseteq \left[n\right]}{\sum }\hat{f}\left(U\right)\cdot \chi_{U}\left(x\right),$$

where [*n*] = {1, 2, ..., *n*} and

$$\chi_{U}\left(x\right)=\underset{i\in U}{\prod }x_{i}$$

are the *parity functions *on variables in *U*. The *Fourier coefficients *$\hat{f}\left(U\right)$ appearing in Equation 2 are given by

(3) $$\hat{f}\left(U\right)=2^{-n}\underset{x\in {\left\{-1,+1\right\}}^{n}}{\sum }f\left(x\right)\cdot \chi_{U}\left(x\right).$$

The number of Fourier coefficients is 2*^n ^*and each takes values in the interval [-1, 1] and is a multiple of 2^-*n*+1^. *Parseval's *theorem can be stated as

(4) $$\underset{U\subseteq \left[n\right]}{\sum }\hat{f}{\left(U\right)}^{2}=1.$$

A particular property that is used later is the following. If *f *does not depend on the variable *i*, then

(5) $$\hat{f}\left(U\right)=0\;\text{if }i\in U.$$

Using this fact, Parseval's theorem implies that for a constant function *f*,

$$|\hat{f}\left(\emptyset \right)|=1\;\text{and}\;\hat{f}\left(U\right)=0\;\text{for all }U\neq \emptyset .$$

Further, if *f *is a (*n*, *k*)-junta, all coefficients *f*(*U*) with |*U*| >*k *are zero, which reduces the maximal number of non-zero coefficients to 2*^k^*. All coefficients are multiples of 2^-*k*+1^, i.e., for some *c *∈ ℤ

(6) $$\hat{f}\left(U\right)=c\cdot 2^{-k+1}\;\text{with}\;|c|\leq 2^{k-1}.$$

Hence, for any non-zero $\hat{f}\left(U\right)$,

(7) $$\underset{U\neq \emptyset }{\min}|\hat{f}\left(U\right)|\;\geq 2^{-k+1}.$$

Spectral learning techniques identify a function or its dependencies from randomly drawn samples by estimating the spectral coefficients. Given a set of samples $X^{m}=\left\{\left(X_{1}^{\prime },\;Y_{1}^{\prime }\right),\;\ldots ,\;\left(X_{m},\;{Y_{m}}^{\prime }\right)\right\}$, an estimator $ĥ\left(U\right)$of the coefficient $\hat{f}\left(U\right)$ is given by

(8) $$ĥ\left(U\right)=\frac{1}{m{\left(1-2\varepsilon \right)}^{|U|+1}}\overset{m}{\underset{i=1}{\sum }}Y_{j}^{\prime }\cdot \chi_{U}\left(X_{j}^{\prime }\right).$$

A similar approach was first proposed in [21] for the noiseless case and can also be used in the presence of noise [22]. It can be shown that

(9) $$E\left\{ĥ\left(U\right)\right\}=\hat{f}\left(U\right),$$

see, for example, [22]. If the number of samples *m *grows, the estimator Equation 8 will converge to its expected value, namely $\hat{f}\left(U\right)$.

### 3.2 Spectral properties of specific regulatory functions

The Boolean functions mentioned in Section 2.3 be categorized according to their *lowness *[6].

**Definition 1**. *A Boolean function f *: {-1, +1}*^n ^*→ {-1, +1}*is τ -low if for any i *∈ var*(f) there exists a set U *⊆ [*n*] *with *0 < |*U*| ≤ *τ such that i *∈ *U and*

$$|\hat{f}\left(U\right)|>0.$$

Clearly any function that is *τ*-low is also *τ′*-low if *τ′ *>*τ*. The notation of lowness allows to define the following families of classes.

**Definition 2**. $C_{\tau }$*is the set of functions that are τ-low*.

In this paper, the focus is on $\left\lceil \frac{2}{3}k\right\rceil$-low and 1-low functions. First, the latter class is considered. All unate functions are 1-low. This follows as

(10) $$|\hat{f}\left(\left\{i\right\}\right)|\;={\text{I}}_{i}\left(f\right),\;\text{if }f\;\text{is unate,}$$

[23], and the fact that for any Boolean function, the influence of an essential variable is larger than zero. Hence, if the *i*th variable of a unate function *f *is essential, the Fourier coefficient $\hat{f}\left(\left\{i\right\}\right)$ is non-zero.

Now the class $C_{\left\lceil \frac{2}{3}k\right\rceil }$ is discussed, first the following definition is needed.

**Definition 3**. *A function f *: {-1, +1}*^n ^*→ {-1, +1} *is mth-order correlation immune if for all U *⊆ [*n*] *with *1 ≤ |*U*| ≤ *m*

$$\hat{f}\left(U\right)=0.$$

Correlation immune functions were considered by Siegenthaler [24] who used a different definition. The definition in terms of the Fourier coefficients as used here is due to Xiao and Massey [25]. These functions are of interest in cryptography, for example, to design combining functions of stream ciphers.

Unbalanced correlation immune functions cannot exist for too large *m *as the next theorem shows.

**Theorem 1 **(Mossel et al. [8]). *Let f *: {-1, +1}*^n ^*→ {-1, +1} *be an unbalanced, mth order correlation immune function. Then *$m\leq \frac{2}{3}\cdot n$.

A similar proposition holds for functions with low average sensitivity.

**Proposition 1**. *Let f *: {-1, +1}*^n ^*→ {-1, +1} *be a mth-order correlation immune function such that *as $\left(f\right)\leq \frac{2}{3}n$, *where ***X **∈ {-1, +1}*^n ^is uniformly distributed. Then *$m\leq \frac{2}{3}\cdot n$.

*Proof*. If *f *is unbalanced, the proposition is true. Suppose *f *is balanced. Assume for contradiction that

(11) $$|\hat{f}\left(U\right)|\;=0\;\text{for }1\leq \;|U|\;\leq m=\frac{2}{3}n.$$

From Parseval's theorem it follows that

$$\begin{array}{lll} \text{as}\left(f\right) & =\underset{U\subseteq \left[n\right]}{\sum }|U|\hat{f}{\left(U\right)}^{2}=\underset{|U|>m}{\sum }|U|\hat{f}{\left(U\right)}^{2}\; & \text{(1)}\\ & >m\underset{U\neq \emptyset }{\sum }\hat{f}{\left(U\right)}^{2}=m\cdot \left(1-\hat{f}{\left(\emptyset \right)}^{2}\right)=\frac{2}{3}n\; & \text{(2)}\\ & \; & \text{(3)} \end{array}$$

which contradicts the assumption of the proposition.   □

**Proposition 2**. *Let f be a function with k *≥ 2 *essential variables (out of n) such that any restriction f′ on k′ of its essential variables, where *1 <*k′ *≤ *k, has an average sensitivity less or equal than *$\frac{2}{3}k^{\prime }$*or is an unbalanced functions (or both). Then f is *$\left\lceil \frac{2}{3}k\right\rceil$-*low*.

*Proof*. First note that if *k *= 2 the proposition is true. Now consider a function with *k *> 2. By assumption there is a variable *i *∈ var(*f*) with a "low" coefficient,

**1 Input**: *, n, d*

**2 Output**: $\tilde{R}$ the essential variables

**3 Global Parameters**: *τ, ϵ*

4 begin

**5 **   $\tilde{R}=\emptyset$;

**6    foreach ***U *⊆ [*n*] *and *1 ≤ *|U| *≤ *τ ***do**

**7 **      $ĥ\left(U\right)\leftarrow {\left(1-2\varepsilon \right)}^{-|U|-1}\cdot m^{-1}\sum_{\left(x\text{,}y\right)\in \chi }y\cdot \chi_{U}\left(x\right)$;

**8       if **$|ĥ\left(U\right)|\;\;\geq 2^{-d}$**then**

**9 **         $\tilde{R}\leftarrow \tilde{R}\cup U$;

10       end

11    end

12 end

**Algorithm 1**: *τ*-NOISY-FOURIER*_d_*

that is *U *∋ *i *and $\left|U\right|\leq \frac{2}{3}k$. Consider the restrictions of *f *to the variable *i *denoted with *f*_-1 _and *f*_+1. _It is straightforward to show that

(12) $$\hat{f}\left(U\right)=\frac{1}{2}\left({\hat{f}}_{+1}\left(U\backslash \left\{i\right\}\right)+{\left(-1\right)}^{\left|\left\{i\right\}\cap U\right|}{\hat{f}}_{-1}\left(U\backslash \left\{i\right\}\right)\right).$$

For variable *j *≠ *i *there is a set *V *∋ *j *and *i *∉ *V *with $\left|V\right|\leq \frac{2}{3}\left(k-1\right)$ such that either ${\hat{f}}_{+1}\left(V\right)\neq 0$ or ${\hat{f}}_{-1}\left(V\right)\neq 0$ Eq. (12) implies that either $\hat{f}\left(V\right)$ or $\hat{f}\left(V\cup \left\{i\right\}\right)$ not equal to zero. In the worst case one has to consider the coefficient $\hat{f}\left(V\cup \left\{i\right\}\right)$. Now note that as |*V *∪ {*i*}| is an integer number

$$|V\cup \{i\}|\leq \left\lfloor \frac{2}{3}(k-1)\right\rfloor +1\leq \left\lceil \frac{2}{3}k\right\rceil .$$

This argument can now be repeated recursively (applying Eq. (12) to *f*_-1 _and *f*_+1_) showing the proposition.   □

### 3.3 The *τ-*NOISY-FOURIER*_d _*algorithm

A simple algorithm to find the essential variables of *τ*-low (*n, k*)-juntas directly follows from Equations 6 and 7. First, all Fourier coefficients up to weight *τ *are estimated. The absolute value of each estimated coefficient ĥ(U) is compared with a threshold. If a coefficient $\hat{f}\left(U\right)$ is non-zero, its absolute value cannot be smaller then 2^-*k*+1^, see Equation 7. Hence, if |ĥ(U)| is larger than 2*^-k^*, the variables corresponding to *U *are classified as essential. The algorithm was given by [6], but they used 2^-*d*-1 ^as threshold (see Line 8).

The following theorem appeared first in [6] but with a different bound.

**Theorem 2**. *Let f be a τ-low *(*n*, *k*)*-junta and*

(13) m≥2⋅22k⋅(1-2ε)-2τ-2 ln2nτδ.

*Then Algorithm *1 *identifies all essential variables with probability *1 - *δ*.

The bound is even true if *ϵ *is only an upper bound on the noise rate. The theorem follows from applying standard Hoeffding bounds. Note that the bound above is different to [6]. If *τ *= 1, the number of samples required to reach a predefined probability of error is smaller by a factor 4. This directly follows from the different threshold used here. If *τ *> 1, it was claimed in [6] that *n^τ ^*can be replaced by *n*. But simulation results of the authors (not shown) contradict this result; hence, we rely here on the weaker result shown in Theorem 2. This issue will be discussed in future work.

### 3.4 Improved algorithms

In the following section, two algorithms are discussed that lead to better numerical results as Algorithm 1 especially for low *k*. The first algorithm is a straight forward modification of the *τ*-NOISY-FOURIER algorithm and is discussed in Section 3.4.1. The second algorithm requires a further assumption on the functions to which it is applied; namely, suppose that *f *is *τ*-low. If a variable of the function *f *is set to a particular fixed value, i.e., -1 or +1, the *restricted *version of *f *is obtained (this will be discussed in more detail later on). Now it has to be assumed that the restricted function is still *τ*-low, i.e., they have to be *recursive τ*-low. While it is possible to define such classes, only unate functions are considered. On the one hand, they naturally fulfill the constraint defined above, as any restriction of a unate function is again a unate function. On the other hand, they seem to be the most important class of functions as discussed earlier. Nevertheless, the following algorithms will be formulated in a way such that it is clear how to apply them for recursive *τ*-low functions.

#### 3.4.1 A modification of the *τ*-NOISY-FOURIER*_d_*

**Figure 1:**
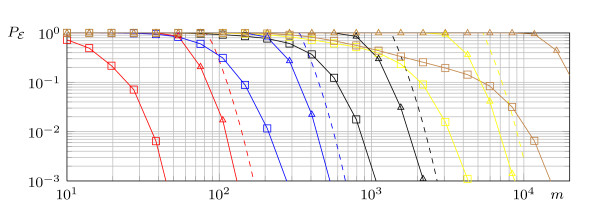
**The average detection error in 10000 trials**: Theoretical bound (dashed), original (triangle), and modified (box) *τ*-NOISY-FOURIER*_d_*, for unate functions with *n *= 500, *ϵ *= 0.05, *d *= *k *= 1 (red), 2 (blue), 3 (black), 4 (yellow), 5 (brown).

Algorithm 1 suffers from a high number of so-called type-2-errors, i.e., it classifies non-essential variables as essential, especially for a small number of samples *m*. Hence, a simple modification is to return only a limited number of essential variables by taking only the variables that correspond to the coefficients with largest absolute value. The algorithm is denoted by *τ *-NOISY-FOURIER^MOD ^and is shown below. The computational complexity of the algorithm increases compared with Algorithm 1. In line 8 $\left({}_{\tau }^{n}\right)$, many spectral coefficients have to be sorted which can be done in roughly *n*^2*τ *^in the worst case [26].^d ^In Figure 1 on page 19, the effect of the modification on the detection error is numerically studied.

#### 3.4.2 The KJUNTA algorithm

The second algorithm is based on the original idea of Mossel et al. [8] who recursively applied their algorithm to restricted functions of the original. While they did for other reasons, a slight modification of their approach can be used to reduce the number of samples needed. The running time of the algorithm is increased by an exponential dependency on *k*.

**1 Input**: *, n, d*

**2 Output**: $\tilde{R}$ the essential variables

**3 Global Parameters**: *τ, ϵ*

4 begin

**5    **$\tilde{R}\leftarrow \emptyset$;

**6    foreach ***U *⊆ [*n*] *and *|*U*| ≤ *τ ***do**

**7 **      $ĥ\left(U\right)\leftarrow {\left(1-2\varepsilon \right)}^{-|U|-1}\cdot m^{-1}\cdot \sum_{\left(x\text{,}y\right)\in X}y\cdot \chi_{U}\left(x\right)$;

8    end

**9 **   $U_{i}:|ĥ\left(U_{1}\right)|\geq |ĥ\left(U_{2}\right)|\geq \cdots \geq |ĥ\left(U_{l}\right)|$   // mod: sorted index;

**10    for ***i *= 1 *to l ***do**

**11       if **$|\tilde{R}|<d$**then **   // mod: limiting condition

**12          if $|ĥ\left(U_{i}\right)|\geq 2^{-d}$ then $\tilde{R}\leftarrow \tilde{R}\cup U_{i}$**;

13       end

14    end

15 end

**Algorithm 2**: *τ *-NOISY-FOURIER^MOD^

To describe the algorithm, some additional definitions are needed. Define a (*n*, *d*) restriction *ρ *= (*ρ*_1_, *ρ*_2_, ..., *ρ_n_*) as a vector of length *n *which consists of symbols in {+1, -1, *}, where the symbol * occurs exactly *d *times. The *restricted *function *f|_ρ _*can be obtained from the function *f *by fixing *d *arguments *x_i _*in the following way. If *ρ_i _*≠ * then *x_i _*= *ρ_i_*. All *x_i _*for *i *such that *ρ_i _*= * are the arguments of *f|_ρ_*; hence, it depends on at most *d *arguments. A vector **x **of length *n *matches if for all *ρ_i _*≠ * it holds that *x_i _*= *ρ_i_*. The restricted samples set $X_{\rho }$ is defined as a subset of  that contains all samples (**x**, y) such that **x **matches the restriction *ρ*, i.e.,

$$X_{\rho }=\langle \left(x,\;y\right)\in X|x\;\text{matches}\;\rho \rangle .$$

The algorithm is now described as follows. Suppose there exists a procedure IDENTIFY that can identify at least one essential variable of a function *f *given a number of samples. If no essential variables exist, i.e., if *f *is constant, the procedure returns the empty set Ø.

Given a (*n*, *k*)-junta *f*, with *k *> 0, and a set *I *⊆ *R *= var(*f*) that contains some essential variables that are already known. Further, assume that there is a restriction *ρ *that fixes exactly the variables in *I*. The function *f|_ρ _*can be either the constant function or depend on some of the variables that are not fixed yet. For the latter case suppose that at least one new variable can be identified, using procedure IDENTIFY. Denote the set of newly identified variables with *I*. Then the procedure is continued with all of the 2^|*I*| ^new restrictions that fix the variables in *I *until all these sub-restrictions will be constant. The resulting algorithm in a recursive form is given as Algorithm 3. Initially, the algorithm is started with KJUNTA(X,n,d), where the global parameters (*τ *= 1, *ϵ*) are fixed.

Most of the algorithm has been explained already. First note that passing *n *as an argument is not necessary, because it is an implicit parameter of the

**1 Input**: *, n, d*

**2 Output**: $\tilde{R}$ the essential variables

**3 Global Parameters**: *τ*, *ϵ*

4 begin

**5 **   $\tilde{R}\leftarrow \emptyset$;

**6 **   I←IDENTIFY(X,d);

**7    if **(*d *> |*I*| > 0) **then**

**8 **      ${\tilde{R}}^{\prime }\leftarrow \emptyset$;

**9       foreach ***restriction ρ ***do**

**10 **         ${\tilde{R}}^{\prime }\leftarrow {\tilde{R}}^{\prime }\cup \text{K}\text{J}\text{UNTA}\;\left(X_{\rho },\;n\;-|I|,\;d\;-|I|\right)$;

11       end

**12 **      $\tilde{R}\leftarrow \text{COMBINE}\left(\tilde{R},\;{\tilde{R}}^{\prime },\;\rho \right)$;

13    end

14 end

**Algorithm 3**: KJUNTA

**1 Input**: , *n*, *d*

**2 Output**: *I *variables found

**3 Global Parameters**: *τ*, *ϵ*

4 begin

**5 **   *I *← ∅;

**6    foreach ***U *⊆ [*n*] *and *|U| ≤ *τ ***do**

**7 **      $ĥ\left(U\right)\leftarrow {\left(1-2\varepsilon \right)}^{-|U|-1}\cdot m^{-1}\cdot \sum_{\left(x,y\right)\in X}y\cdot \chi_{U}\left(x\right)$;

8    end

**9 **   $M\leftarrow \arg\max_{U:0<|U|\leq \tau }|\hat{h}(U)|$;

**10    if **$\left(\text{CONST}\;\text{(}\hat{h}(M),\;\hat{h}(\emptyset ),\;d)=true\right)$**then ***I *← *M *;

11 end

**Algorithm 4**: IDENTIFY

samples. Further comments should be given to the line 9. The *foreach loop *is executed for each of the 2^|*I*| ^possible restrictions of the variables contained in *I*. For each restriction, the corresponding restricted sample set is calculated and passed in a new call to KJUNTA. Each of these calls runs on smaller problems, namely finding variables of a (*n - *|*I*|, *d *- |*I*|)-junta. Notably, each of these runs is independent of the others. The variables found are then combined with $\tilde{R}$ in line 11 using the procedure COMBINE. This is not just a union of sets since one has to take care about the labeling of the variables. For example, if $\tilde{R}=\left\{1\right\}$, and a subsequent call of KJUNTA returns variables joined to ${\tilde{R}}^{\prime }=\left\{1,3\right\}$, combining both leads to $\tilde{R}=\left\{1,2,4\right\}$.

**The **IDENTIFY** procedure **The question remains how to identify some of the essential variables or how to decide whether the function is constant. For *τ*-low functions, it is sufficient to estimate all coefficients $\hat{f}\left(U\right)$ with |*U*| ≤ *τ*. In [7], it was proposed to search for the first coefficient that is above a certain threshold. The approach here is different. In particular, all coefficients with weight less or equal *τ *are computed. The coefficient with the maximum absolute value is compared with the zero coefficient to distinguish between a constant and a non-constant function. How this can be done is discussed below. The resulting algorithm is formulated in terms of Algorithm 4 on page 12. In line 8, the procedure CONST is called which tries to distinguish between a constant function and a non-constant function. If a non-constant function is found, the variables in *M *are returned, otherwise the empty set.

**The **CONST** procedure **In the following it is discussed how a constant function can be distinguished from a non-constant function, given that the function depends on not more than *k *variables. This is done based on the zero coefficient $\hat{f}\left(\emptyset \right)$ and the coefficient with the largest absolute value, denoted by $\hat{f}\left(M\right)$. Note that if and only if *f *is constant, $|\hat{f}\left(\emptyset \right)|=1$ and $\hat{f}\left(U\right)=0$ for any set *U *≠ ∅ by Parseval's theorem. If *f *is non-constant, $|\hat{f}\left(\emptyset \right)|<1$ and there exists at least one coefficient with $|\hat{f}\left(U\right)|>0$ for some *U*; hence, it follows that $|\hat{f}\left(M\right)|>0$.

To distinguish between a constant and a non-constant function different procedures exist. The most simple one was proposed by Mossel et al. which will be denoted by CONST1. There, if $|ĥ\left(\emptyset \right)|>1-2^{-d}$ or $|ĥ\left(M\right)|<2^{-d}$, the function is declared as constant.

For small *d*, a better procedure, that requires less samples, exists. It is denoted by CONST2. Given the 2-tuple (ĥ(∅),ĥ(M)) compute the--in Euclidean distance-- closest tuple (*α*, *β*) such that *α *< 1, *β *> 0 are multiples of 2^-*d*+1^. Hence, the function is declared as constant if

$$\text{dist}\;\left(\left(ĥ\left(\emptyset \right),\;ĥ\left(M\right)\right),\;\left(1,\;0\right)\right)<\text{dist}\;\left(\left(ĥ\left(\emptyset \right),\;ĥ\left(M\right)\right),\;\left(\alpha ,\;\beta \right)\right),$$

where dist (·,·) denotes the Euclidean distance.

**A note on the computational complexity **As mentioned, Algorithm 3 has an increased complexity compared with Algorithm 1. In the worst case, the algorithm is called 2*^k ^*times, but clearly each time on a smaller problem. If it is assumed that $ĥ\left(U\right)$ can be computed in time O(n⋅m), the algorithm runs in $O\left({\text{2}}^{k}\cdot n^{\text{2}}\cdot m\right)$ for 1-low functions. Obviously for constant *k*, this reduces to $O\left(n^{\text{2}}\cdot m\right)$.

### 3.5 Simulation results for unate networks

To compare the performance of the different algorithms, the following procedure is used. Suppose a BF *f *is chosen uniformly at random from a class $ℱ\subseteq {ℱ}^{n}$ of *n*-ary *τ*-low functions, where *τ *and *n *are known. For the functions *f*, a set of *m *noisy state-transitions $X^{m}=\left\{\left(X_{l}^{\prime },\;Y_{l}^{\prime }\right)|l=1..m\right\}$ is generated as described in Section 2.2. The noise rate is fixed to *ϵ *= 0.05.

The most important indicator is the probability of a detection error. Define  as the event $\left\{\tilde{R}\neq \text{var}\left(f\right)\right\}$ where $\tilde{R}$ is the *detected variable set*. The detection error probability

$$P_{ℰ}=\text{Pr}\left\{\tilde{R}\neq \text{var}\left(f\right)\right\}$$

is a prior indicator on the algorithm's performance. It should be mentioned that if there exists a function *f *such that var(*f*) >*d*, the detection error probability $P_{ℰ}$ does not vanish, even for large *m*.

Further evaluation criteria that are used in Section 3.5.3 are the *precision rate ρ *and the *false-negative rate β*. In the present context, the precision rate is defined as the conditional probability that a detected variable is indeed an essential variable, i.e.,

$$\rho =\text{Pr}\left\{i\in \text{var}\left(f\right)|i\in \tilde{R}\right\}.$$

An equivalent way of stating that matter is that a predicted edge *e *is in *E*, where *G*(*V*, *E*) is the associated graph of the network. The false-negative rate is defined as the conditional probability that an essential variable is not detected as being essential,

$$\beta =\text{Pr}\left\{i\notin \tilde{R}|i\in \text{var}\left(f\right)\right\}.$$

In a network, this can be interpreted as the fraction of edges that have not been detected. The definitions above are consistent with Zhao et al. [27] who defined the type-1-error as the event that a node *i *is classified as a controlling node of some node *j *although this is not the case. Consequently the type-2-error is defined as the event $\left\{i\notin \tilde{R}|i\in \text{var}\left(f\right)\right\}$.

#### 3.5.1 *τ*-NOISY-FOURIER*_d _*versus $\tau -{\text{N}\text{OISY}-\text{F}\text{OURIER}}_{d}^{mod}$

**Figure 2:**
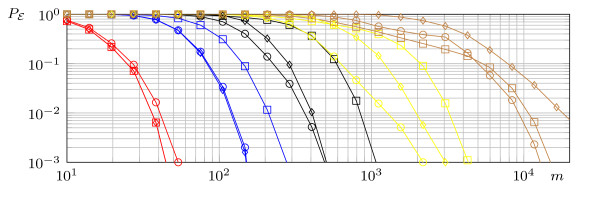
**The average detection error in 10,000 trials**: $\tau -\text{N}\text{OISY}-\text{F}{\text{OURIER}}_{d}^{\text{mod}}$ (box) and KJUNTA with CONST1 (circle) and CONST2 (diamond) procedure, unate functions (*n *= 500, *ϵ *= 0.05, *d *= *k *= 1 (red), 2 (blue), 3 (black), 4 (yellow), 5 (brown).

First, the modified version of the *τ*-NOISY-FOURIER*_d _*algorithm is compared with the original algorithm. In 10,000 independent experiments, unate functions with exactly *k *essential variables are randomly drawn. The parameter *d *is always set to *k*. The results are presented in Figure 2, further the upper bounds on the detection error probability (Theorem 2) are shown. As promised $\tau -\text{N}\text{OISY}-\text{F}{\text{OURIER}}_{d}^{\text{mod}}$ outperforms the original algorithm.

#### 3.5.2 $\tau -{\text{N}\text{OISY}-\text{F}\text{OURIER}}_{d}^{mod}$ versus KJUNTA

Again a subset of unate functions with exactly *k *essential variables is used to compare the $\tau -\text{N}\text{OISY}-\text{F}{\text{OURIER}}_{d}^{\text{mod}}$ algorithm with the KJUNTA algorithm. The parameter *d *is always set to *k*. The results are shown in Figure 2. For functions with a low number of essential variables, the procedure CONST1 outperforms the *τ*-NOISY-FOURIER*_d _*algorithm. But the better performance vanishes with an increasing number of variables.

#### 3.5.3 *τ*-NOISY-FOURIER*_d _*versus KJUNTA on an *E. coli *network

**Figure 3:**
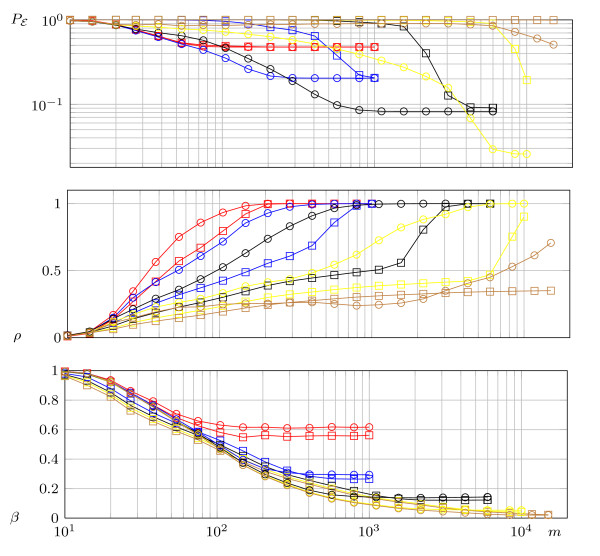
**Simulation results for the modified **$\tau -\text{N}\text{OISY}-{\text{F}\text{OURIER}}_{d}^{mod}$** (box) and KJUNTA with the CONST1 (circle) procedure applied on the regulatory functions of a network of *E. coli*, see text**. (*n *= 583, *ϵ *= 0.05, *d *= *k *= 1 (red), 2 (blue), 3 (black), 4 (yellow), 5 (brown).

In this simulation, the functions are chosen from the regulatory functions of the control network of the *E. coli *metabolism [9]. This set includes functions with a different number of essential variables. Further, also some constant functions are included and some functions occur several times. Each function *f *has 583 possible arguments but depends on not more than eight variables. The functions distribution on essential variables is given in Table 1 and is equivalent to the in-degree distribution of the corresponding network.^e ^The results in Figure 3 are obtained by applying the algorithms to each function in the set, this experiment is performed 100 times.

**Table 1:** In-degree distribution of the Boolean network (see text).

|var(*f*)|	0	1	2	3	4	5	6	7	8
#	12	293	159	66	38	9	4	0	2

**Remarkable results: **In the previous simulations, the parameter *d *is always set to *k*. Further only functions with *exactly k *essential variables are chosen. Here, the parameter *d *is usually smaller than *k*, which implies that not all variables can be found. Only variables with influence large or equal 2^-*d *^can be detected. This is implied by Equations 10 and 7. On the other hand, even if *d *<*k *for some function *f*, the algorithm can possibly detect some of the essential variables of *f*.

## 4 Conclusion

In this paper, the problem to detect controlling nodes in Boolean networks is discussed. Boolean functions that are relevant for modeling genetic networks seem to belong to classes of functions for which spectral-based algorithms provide an efficient solution--both, in computational complexity and data needed. Especially the algorithms for unate functions are highly efficient in both running time and the number of samples needed to identify controlling nodes. Further analytical bounds on the probability of a detection error can be stated.

If the samples are chosen according to a uniform distribution, the results are promising. Applying the methods to the *E*. *coli *control network, with 583 nodes, shows that using approximately 200 samples, it is possible to find nearly 40% of all edges in the network with a precision rate close to one. On the other hand, a wrong selection of the parameter *d *can have a dramatic effect on the precision. For example, if under the same conditions *d *= 4 is chosen, the precision will drop below 0.5. Fortunately, the choice of the parameter can be guided by the available analytical bounds of the detection error probability. The latter is dominated by the probability that the estimator ĥ({i}) will deviate from $\hat{f}\left(\left\{i\right\}\right)$ by more than +/- 2^-*d*^. But this also determines the precision of the algorithm. Suppose that 200 samples are obtained from the *E. coli *network. The analytical bounds shown in Figure 1 suggest to choose *d *= 1 which indeed leads to a high precision (see Figure 3).

Clearly, our assumption of uniformly distributed samples is too optimistic. Fortunately, known results from PAC learning [6] show that it is possible to use similar algorithms for product distributed samples, i.e., in a random vector **X **each *X_i _*is chosen independently of the others with a certain probability such that $-\text{1}<E\left\{X_{i}\right\}=\mu_{i}<\text{1}$. But there is a major problem: If *μ*_max _= max_1≤*i*≤*n *_|*μ_i_*| gets closer to 1, the number of samples needed will increase with roughly (1 - *μ*_max_)^-2*k*^. In unate networks, this coincides with the fact that the influences of the variables can become very small. Hence, further investigations in this direction are necessary. This would be a major step toward the application of spectral algorithms in a real-world scenario.

## 5 Competing interests

The authors declare that they have no competing interests.

## Endnotes

^a^The theoretical analysis requires the noise level to be bounded below a small value. ^b^This will be defined more precisely later. ^c^A function is unbalanced if the number of +1 and -1 in the truth table is different. ^d^Using a better implementation as Algorithm 2, this can be reduced to 2*τ *log *N*. ^e^The detailed table of the used functions can be found in the supplementary material.
